# CenhANCER: a comprehensive cancer enhancer database for primary tissues and cell lines

**DOI:** 10.1093/database/baad022

**Published:** 2023-05-18

**Authors:** Zhi-Hui Luo, Meng-Wei Shi, Yuan Zhang, Dan-Yang Wang, Yi-Bo Tong, Xue-Ling Pan, ShanShan Cheng

**Affiliations:** Department of Epidemiology and Biostatistics, Ministry of Education Key Laboratory of Environment and Health, School of Public Health, Tongji Medical College, Huazhong University of Science and Technology, 13 Hangkong Road, Wuhan, Hubei 430030, P.R. China; Hubei Hongshan Laboratory, College of Biomedicine and Health, Huazhong Agricultural University, No. 1, Shizishan Street, Wuhan, Hubei 430070, China; Hubei Key Laboratory of Agricultural Bioinformatics, College of Life Science and Technology, Huazhong Agricultural University, No. 1, Shizishan Street, Wuhan, Hubei 430070, China; Shenzhen Institute of Nutrition and Health, Huazhong Agricultural University, 97 Buxin Road, Shenzhen 518000, China; Shenzhen Branch, Guangdong Laboratory for Lingnan Modern Agriculture, Genome Analysis Laboratory of the Ministry of Agriculture, Agricultural Genomics Institute at Shenzhen, Chinese Academy of Agricultural Sciences, 97 Buxin Road, Shenzhen 518000, China; Hubei Hongshan Laboratory, College of Biomedicine and Health, Huazhong Agricultural University, No. 1, Shizishan Street, Wuhan, Hubei 430070, China; Hubei Key Laboratory of Agricultural Bioinformatics, College of Life Science and Technology, Huazhong Agricultural University, No. 1, Shizishan Street, Wuhan, Hubei 430070, China; Shenzhen Institute of Nutrition and Health, Huazhong Agricultural University, 97 Buxin Road, Shenzhen 518000, China; Shenzhen Branch, Guangdong Laboratory for Lingnan Modern Agriculture, Genome Analysis Laboratory of the Ministry of Agriculture, Agricultural Genomics Institute at Shenzhen, Chinese Academy of Agricultural Sciences, 97 Buxin Road, Shenzhen 518000, China; Hubei Hongshan Laboratory, College of Biomedicine and Health, Huazhong Agricultural University, No. 1, Shizishan Street, Wuhan, Hubei 430070, China; Hubei Key Laboratory of Agricultural Bioinformatics, College of Life Science and Technology, Huazhong Agricultural University, No. 1, Shizishan Street, Wuhan, Hubei 430070, China; Hubei Hongshan Laboratory, College of Biomedicine and Health, Huazhong Agricultural University, No. 1, Shizishan Street, Wuhan, Hubei 430070, China; Hubei Key Laboratory of Agricultural Bioinformatics, College of Life Science and Technology, Huazhong Agricultural University, No. 1, Shizishan Street, Wuhan, Hubei 430070, China; Hubei Hongshan Laboratory, College of Biomedicine and Health, Huazhong Agricultural University, No. 1, Shizishan Street, Wuhan, Hubei 430070, China; Hubei Key Laboratory of Agricultural Bioinformatics, College of Life Science and Technology, Huazhong Agricultural University, No. 1, Shizishan Street, Wuhan, Hubei 430070, China; Shenzhen Institute of Nutrition and Health, Huazhong Agricultural University, 97 Buxin Road, Shenzhen 518000, China; Shenzhen Branch, Guangdong Laboratory for Lingnan Modern Agriculture, Genome Analysis Laboratory of the Ministry of Agriculture, Agricultural Genomics Institute at Shenzhen, Chinese Academy of Agricultural Sciences, 97 Buxin Road, Shenzhen 518000, China; Department of Epidemiology and Biostatistics, Ministry of Education Key Laboratory of Environment and Health, School of Public Health, Tongji Medical College, Huazhong University of Science and Technology, 13 Hangkong Road, Wuhan, Hubei 430030, P.R. China

## Abstract

Enhancers, which are key tumorigenic factors with wide applications for subtyping, diagnosis and treatment of cancer, are attracting increasing attention in the cancer research. However, systematic analysis of cancer enhancers poses a challenge due to the lack of integrative data resources, especially those from tumor primary tissues. To provide a comprehensive enhancer profile across cancer types, we developed a cancer enhancer database CenhANCER by curating public resources including all the public H3K27ac ChIP-Seq data from 805 primary tissue samples and 671 cell line samples across 41 cancer types. In total, 57 029 408 typical enhancers, 978 411 super-enhancers and 226 726 enriched transcription factors were identified. We annotated the super-enhancers with chromatin accessibility regions, cancer expression quantitative trait loci (eQTLs), genotype-tissue expression eQTLs and genome-wide association study risk single nucleotide polymorphisms (SNPs) for further functional analysis. The identified enhancers were highly consistent with accessible chromatin regions in the corresponding cancer types, and all the 10 super-enhancer regions identified from one colorectal cancer study were recapitulated in our CenhANCER, both of which testified the high quality of our data. CenhANCER with high-quality cancer enhancer candidates and transcription factors that are potential therapeutic targets across multiple cancer types provides a credible resource for single cancer analysis and for comparative studies of various cancer types.

**Database URL** http://cenhancer.chenzxlab.cn/

## Introduction

Enhancers, which are short DNA regions commonly marked by the histone modification H3K27 acetylation (H3K27ac), can positively regulate spatiotemporal gene expression (1) and have been widely explored in a variety of cancer types (2). For example, enhancers display distinct profiles in four histotypes of ovarian cancer, which indicates their application value in cancer subtyping (3). The activity of H3K27ac is correlated with clinical characteristics of acute myeloid leukemia, suggesting its prognostic application in cancer (4). A therapeutic strategy based on enhancer-blocking bromodomain inhibitors is devised for colorectal cancer, implying its therapeutic potential (5). Furthermore, recent studies have revealed the *cis*-regulatory effect of enhancers in disease development with the aid of risk single nucleotide polymorphisms (SNPs) identified from genome-wide association studies (GWASs) (6, 7). All these findings show the key role of enhancers in cancer research.

Although cancer onset and progression are widely acknowledged to be associated with enhancer activities, the functions and mechanisms of regulation of most enhancers remain elusive due to the limited integrative data sources and the challenging nature of functional analysis. Although H3K27ac biosamples have been rapidly accumulated in recent years, there is no large-scale pan-cancer project with this type of data, while the existing large epigenomic projects such as ENCODE and Roadmap mainly involve normal tissues and cell lines (8).

To provide comprehensive cancer enhancer resources, we established a user-friendly database CenhANCER (http://cenhancer.chenzxlab.cn/) by manually curating all public H3K27ac ChIP-Seq data of cancer primary tissues and cell lines from Gene Expression Omnibus (GEO) database, and these data were reliable for enhancer identification (9). Typical enhancers, super-enhancers and transcription factors (TFs) were identified by standard pipelines. To annotate enhancers and their association with cancer, we integrated TCGA ATAC-seq (10), cancer expression quantitative trait locus (eQTL) (11), genotype-tissue expression (GTEx) eQTL (12) and GWAS risk SNP data (13) with the enhancers. CenhANCER is thus a database that consolidates high-quality cancer enhancer candidates and TFs that are potential therapeutic targets, a resource that will be highly valuable for cancer research.

## Materials and methods

**Figure 1.**
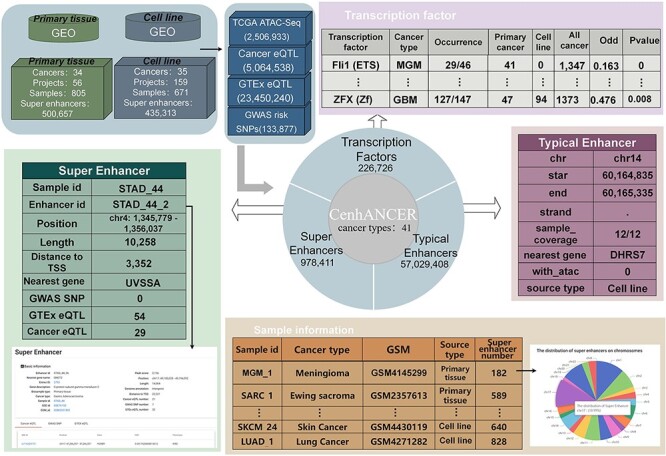
CenhANCER database structure and content. The main data include ChIP-Seq data of H3K27ac samples covering cancer primary tissues and cell line samples. ATAC-seq ACRs, cancer eQTLs, GTEx eQTLs and GWAS risk SNPs are supplementary annotation for enhancers. CenhANCER consist of four major parts, namely, sample information, super-enhancers, typical enhancers and TFs.

The database was established through data integration, enhancer identification, enhancer annotation and TF enrichment (Figure 1).

### Data sources

We collected cancer-related ChIP-Seq samples from GEO database by a text-mining pipeline we developed. Notably, all the information from National Center for Biotechnology Information was obtained by using the ‘Bio.Entrez’ python package. First, we retrieved 1203 study projects (GSE accessions) from GEO with ‘(H3K27ac[All Fields] AND (“neoplasms”[MeSH Terms] OR cancer[All Fields])) AND “Homo sapiens”[Organism] AND “gse”[Filter]’ as keyword. Then, we extracted 51 748 samples from these projects. Second, the metadata containing sample attribute descriptive information in the form of free text were extracted from the xml files obtained in the Sequence Read Archive (SRA). Third, to ensure that the samples were H3K27ac ChIP-seq data, these samples whose attributes contained no ‘ChIP-Seq’ or ‘H3K27ac’ were filtered. The primary tissue samples with their attributes containing ‘cell line’ were filtered out. Then, we manually curated the samples without any chemical treatment from 2317 samples of 251 projects and further integrated their corresponding input and adjacent samples. We limited cell line samples to those with attributes containing ‘cell line’ or ‘cell_line’ and ensured that the selected samples were not treated with drugs. Finally, we obtained 805 primary cancer tissues and 671 cancer cell lines across 41 cancer types. Each cancer type is manually assigned an abbreviation (Supplementary Table S1).

### Detection of typical enhancers and super-enhancers

The raw fastq data were extracted from SRA files with fastq-dump (14), and their quality control was performed using fastqc (15). The low-quality reads were excluded. Then, adapters were removed by fastp with default parameters (16). Clean reads were mapped to the human reference genome (GRCh38, http://ftp.ebi.ac.uk/pub/databases/gencode/Gencode_human/release_38/GRCh38.primary_assembly.genome.fa.gz). We processed single-end data by bowtie (version 1.2.1) (17) with the parameter ‘--sensitive’ and paired-end data by bowtie2 (version 2.3.3) (18) with parameter ‘--chunkmbs 320 -m 1 --best’. Duplicate reads were removed by Picard.

Typical enhancers were identified from narrow peaks called by macs2 with parameters ‘--nomodel --keep-dup all -p 1E-5 --extsize 147’. We removed the peaks mapped to mitochondria and decoyed contigs. The peaks in the ENCODE hg38 blacklist were also excluded. Narrow peaks were handled according to Kundaje’s pipeline (https://github.com/ENCODE-DCC/chip-seq-pipeline2). All the narrow peaks were normalized to a fixed width of 500 bp by extending 250 bp from the peak center to both upstream and downstream. All the narrow peaks in one cancer type were merged to obtain a cancer-specific peak set. Specifically, in the merging process, two overlapped peaks from two independent samples were merged into one, the peak with a higher score was retained and all the peaks were subjected to pairwise merging to obtain one peak set. Then, the peaks of another sample were merged into the previously merged peak set. After obtaining the overall set of one cancer, we intersected the narrow peaks of all the samples with the overall set. Only the peaks overlapped with those from at least two samples were identified as a reproducible cancer-associated enhancer.

Super-enhancers were identified from each sample by Rank Ordering of Super-Enhancers with the parameters ‘-t 2500 -s 12500’ based on the peaks and the mapped bam file (19).

### Overlaps of the typical enhancer and accessible chromatin region

The accessible chromatin regions (ACRs, ATAC-seq peak) of 23 cancer types were downloaded from a recent TCGA publication (10). Of these 23 cancers, 17 cancers were overlapped with those from our CenhANCER. The ACRs were intersected with the cancer-specific enhancers by ‘bedtools intersect’ (https://bedtools.readthedocs.io/en/latest/) with default parameters.

### Risk SNP–associated super-enhancers

We downloaded the known GWAS SNPs from the European Bioinformatics Institute GWAS catalog (13). We normalized the phenotype of each disease with our previously developed tool pyMeSHSim (20). To obtain the cancer terms, we filtered the terms that did not belong to ‘C04 (neoplasms)’. Finally, a total of 366 GWAS projects of cancer were obtained. Then, we retrieved the lead SNPs from these projects and added SNPs with linkage disequilibrium (LD) *r*^2^ > 0.8 to the GWAS lead SNPs. This LD information was obtained from the R package ‘haploR’ with the embeded data from the haploreg website (21). In addition, SNP mutations for cancer types were downloaded from TCGA by TCGAbiolinks (22). All the SNPs were mapped to the super-enhancers by ‘bedtools intersect’.

### GTEx eQTL–associated super-enhancers

The eQTLs obtained from the GTEx project were linked to the cancer super-enhancers and their target genes. The *.signif_variant_gene_pairs.txt.gz files were downloaded from GTEx database (https://gtexportal.org/home/datasets). We intersected the SNPs of eQTL with each super-enhancer with bedtools. Only significant eQTLs (*P*-value < 0.05) were retained for subsequent analysis.

### TF enrichment with typical enhancers

For each sample, binding motifs of TFs were enriched with the corresponding typical enhancers, while transcriptional start sites (TSS) ± 2 kb were removed to exclude an effect from the nucleosome-free regions. The TF binding motifs were identified using the HOMER script (findMotifsGenome.pl) with the parameter of ‘-size 600’ (23). The typical enhancer peaks excluding regions overlapping with TSS ± 2 kb were used as target sequences. An approximately same number of sequences as the number of target sequences will be randomly picked from the genome as background sequences. Then, a hypergeometric test was conducted for occurrences of each TF motif in the target and background sequences (1),


(1) $$P_{hyper} = {\rm{\;}}\mathop \sum \limits^{{min}\left( {T,{\rm{\;}}t + b} \right)}_{i = t} \frac{{\left( {\begin{array}{*{20}{c}}
{t + b}\\
i
\end{array}} \right)\left( {\begin{array}{*{20}{c}}
{T + B - t - b}\\
{T - i}
\end{array}} \right)}}{{\left( {\begin{array}{*{20}{c}}
{T + B}\\
T
\end{array}} \right)}}$$

where *T* and *B* are the number of target and background sequences; *t* and *b* are the subset of target and background sequences containing at least one occurrence of the motif; $i$ is the number of observed sequences containing the motif. ${p_{hyper}}$ is the *P*-value of the hypergeometric test.

Then, HOMER applied the Benjamini–Hochberg false discovery rate method to adjust the *P*-values of all TFs (embedded in HOMER) in each sample. Significantly enriched TF binding motifs were selected based on a *P*-adj < 0.05. Then, the frequency of the TFs enriched from the samples of each cancer type was summarized. We estimated the odds ratio of each TF in each cancer type through Fisher’s exact test.

## Results

### Data summary

Several enhancer-related databases had been reported in recent research studies, and the difference between CenhANCER and these databases is summarized in Table 1. CancerEnd is a cancer-associated database based on expressed enhancer candidates rather than histone modifications (24). OncoBase provided the relationship between cancer somatic mutations and regulatory elements, which were mainly recognized in normal samples (25). OncoCis uncovered somatic mutations located in *cis*-regulatory elements within limited cancer cell line data (26). ENdb and DiseaseEnhancer contained manually collected and experimentally verified disease-associated high-quality enhancers, which makes their total records far less than databases based on high-throughput data (27, 28). SEdb and SEanalysis were two comprehensive web platforms for super-enhancers mainly from normal ChIP-Seq samples (29, 30). To the best of our knowledge, there is still no comprehensive enhancer analysis for all cancer types, due to limited data and the lack of an integrated data source. Here, we provided the first and the largest cancer enhancer resource of ChIP-Seq data from tumor primary tissues and cell lines.

**Table 1.** Comparison between CenhANCER and relevant databases

Database	Data source	Expression enhancer	Typical enhancer	Super-enhancer	ChIP-Seq
CenhANCER	Tumor tissue/cell lines	No	Yes	Yes	Yes
CancerEnD	Tumor tissue	Yes	No	No	No
OncoBase	Normal tissue/cell lines	No	Yes	Yes	Yes
OncoCis	Cell lines	No	Yes	Yes	Yes
ENdb	Normal tissue/cell lines	No	Yes	Yes	Yes
DiseaseEnhancer	Literatures	No	Yes	Yes	No
SEdb	Normal tissue/cell lines	No	No	Yes	Yes
SEanalysis	Normal tissue/cell lines	No	No	Yes	Yes

**Figure 2.**
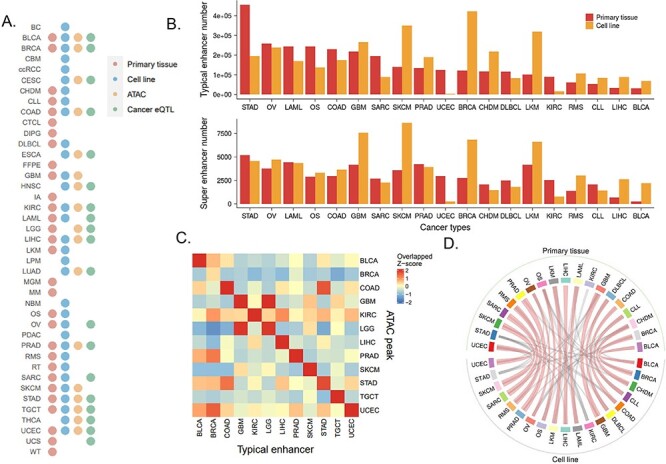
Overview of cancer typical enhancers and super-enhancers. (A) Distribution of samples of primary tissue H3K27ac, cell line H3K27ac, cancer eQTL and primary tissue ATAC-Seq in cancer types. (B) The upper panel exhibits the distribution of reproducible typical enhancer number in cancer types, and the lower panel displays the distribution of merged super-enhancer number in cancer types. (C) The heatmap of the overlap between the primary tissue typical enhancer and the primary tissue ATAC-Seq ACRs. (D) Circos plot of the typical enhancer overlap between primary tissue and cell lines. Each tissue is linked to the top three overlapped cell lines, and the thickened linkage line indicates the same cancer type shared by tissue and cell line.

CenhANCER covered 805 primary tissue samples across 34 cancer types and 671 cell line samples across 35 cancer types. Specifically, it included 2 506 933 ATAC-Seq ACRs across 17 cancers, 5 064 538 cancer eQTLs across 19 cancers, 23 450 240 GTEx eQTLs and 133 877 GWAS risk SNPs (Figure 1, Figure 2A). In total, we identified 57 029 408 typical enhancers, 978 411 super-enhancers (Figure 2B) and 226 726 enriched TFs.

To assess the quality of our identified enhancers, we examined the consistency between the typical enhancers and the ATAC-Seq ACRs, since the ACRs were highly correlated with enhancer distribution (31,32). Specifically, the typical enhancers for each cancer type were intersected with the corresponding ATAC-Seq ACRs. For both primary tissues and cell lines, the overlap between the typical enhancers and the corresponding ATAC-Seq ACRs in the same cancer was higher than that in different cancers (Figure 2C, Figure S1). Interestingly, the typical enhancers covering more samples had a higher overlap with the ACRs for both primary tissues and cell lines (Figure S2A and B). Furthermore, an overlap between the primary tissue enhancers and the cell line enhancers was higher in the same cancer than in different cancers (Figure 2D). All these results indicated a high quality of the typical enhancers in CenhANCER.

### Database access

We designed a user-friendly web platform for data exploration. The users can visit six functional pages including ‘Browser’, ‘SuperEnhancer’, ‘TypicalEnhancer’, ‘TranscriptionFactor’, ‘Download’ and ‘Help’ through the navigation bar in the home page (Figure S3A–F). On the ‘Browser’ page, users can access any sample by selecting one cancer type on the left menu. After clicking one sample, more detailed information will be provided (Figure S3A and B). The summarized super-enhancers can be searched through their loci or nearest genes on the ‘SuperEnhancer’ page (Figure S3C). After clicking one super-enhancer, the users can get its corresponding annotation information (Figure S3D). The reproducible cancer typical enhancer set, categorized by cancer type and data type, is provided on the ‘TypicalEnhancer’ page (Figure S3E). The ‘TranscriptionFactor’ page provides information including TF’s name, occurrence number in a certain cancer and the odds ratio. Only TFs with *P* < 0.01 are taken into account (Figure S3F). All data in CenhANCER can be downloaded on the ‘Download’ page. A detailed tutorial usage is available on the ‘Help’ page.

### Usage example

**Figure 3.**
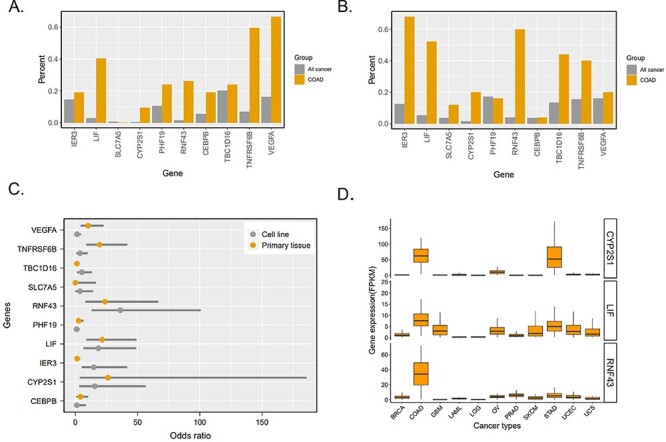
Analysis of 10 gene-related super-enhancers in colorectal cancer. (A) Percentage of 10 gene-related super-enhancers detected in primary tissue samples. ‘All cancer’ indicates cancer types excluding COAD. (B) Percentage of 10 gene-related super-enhancers detected in cell line samples. ‘All cancer’ indicates cancer types excluding COAD. (C) Ten genes’ odds ratio of super-enhancers detected in colorectal cancer to those detected in all other cancers. (D) Three genes’ expression (fragments per kilobase of exon per million mapped fragments, FPKM) in different cancer types according to TCGA RNA-Seq.

To illustrate the usage of CenhANCER in cancer enhancer exploration, we provide an example of super-enhancer analysis. A study of colorectal cancer (32) whose data have not been released is not covered by CenhANCER. In this study, *IER3*, *LIF*, *SLC7A5*, *CYP2S1*, *PHF19*, *RNF43*, *CEBPB*, *TBC1D16*, *TNFRSF6B* and *VEGFA* have been identified as variant super-enhancers loci–associated genes. We retrieve these genes in the ‘SE’ page via the ‘Search by gene’ with ‘Source’ option set as ‘None’, ‘Cancer’ option as ‘None’ or ‘COAD’ (colorectal cancer) and ‘Gene’ option as the gene symbol. Accordingly, we obtained the related super-enhancer loci in colorectal cancer (CRC) and all cancer samples. Ten out of 10 genes (100%) reported in the study are successfully detected in our database. The occurrence frequency of related super-enhancers in CRC is higher than that in other cancers, and this result is consistent for both primary tissue and cell line samples (Figure 3A and B). These findings suggest the high consistency of our data with the unpublished data. The *RNF43-, CYP2S1-* and *LIF*-related super-enhancers are shown as colorectal cancer–specific super-enhancers since the odds ratio of these three gene-related super-enhancers are >20 (*P* < 0.05) in primary tissues and >15 in cell lines (*P* < 0.05) (Figure 3C). To validate these three genes, we downloaded the cancer gene expression data by TCGAbiolinks (22) and explored the expression of these these genes in 11 cancers, which had corresponding H3K27ac ChIP-Seq data in CenhANCER. Interestingly, COAD was the cancer type where *RNF43*, *CYP2S1* and *LIF* showed the highest expression (Figure 3D), suggesting that these three genes’ cancer-specific functions might be attributed to their adjacent super-enhancers. Furthermore, the super-enhancers of *CYP2S1* were also stomach adenocarcinoma (STAD)–specific (Figures S4 and S5). As expected, the *CYP2S1* exhibited a higher gene expression in STAD than in other cancers.

To further verify the target genes that the super-enhancers regulate, we retrieved the eQTLs and SNPs located in super-enhancer regions related to the above-mentioned 10 genes. When a gene’s eQTL or SNP is overlapped with an enhancer region of the same gene, it is highly possible for the enhancer to regulate this gene (10, 33). Finally, we obtained four *TNFRSF6B*-related GWAS risk SNPs in the gene’s adjacent super-enhancer regions from the ‘colorectal cancer’ phenotype (Supplementary Table S2). We obtained 34 GTEx eQTL SNPs in the super-enhancer regions of *LIF*, *PHF19*, *TNFRSF6B* and *CYP2S1* (Supplementary Table S3) from ‘Colon Sigmoid’ or ‘Colon Transverse’ tissues. These results indicated that there might be a regulatory connection between these genes and their adjacent super-enhancers. It is worth noting that two (*LIF* and *CYP2S1*) out of the three colorectal cancer–specific genes aforementioned were identified from eQTLs and SNPs analyses. These results further confirmed that the cancer-specific functions might be attributed to their adjacent super-enhancers. All the aforementioned analyses suggest the in-depth applicability of CenhANCER in cancer research.

## Discussion

CenhANCER is a comprehensive open resource to provide cancer enhancer profiles including typical enhancers, super-enhancers and potential TFs interacting with the enhancers. Convenient retrieval interface has been developed for enhancers, samples and cancer types. All processed data have been integrated for download. CenhANCER is the first ChIP-Seq data-focused cancer enhancer database, covering a large number of primary tissues and cell lines. It also integrated TCGA ATAC-Seq ACRs, GTEx eQTLs, cancer eQTLs and GWAS SNPs as supplementary function annotation. CenhANCER utilizes a semi-auto data collection method, making it easy to update its dataset. Since enhancers exhibit strong regulation function in cancer biology, more and more data will be generated. The cancer samples in our database are updated in real time, and it will provide valuable epigenetic information for both experimental and computational researchers.

## Supplementary Material

baad022_Supp

## Code availability

All codes used for analysis and plotting are available at https://github.com/luozhhub/chip-seq-pyflow.
