# Supplementary material for: CenhANCER: a comprehensive cancer enhancer database for primary tissues and cell lines
Source: Database (Oxford). 2023 May 18;2023:baad022. doi: 10.1093/database/baad022 (PMC10198702; doi:10.1093/database/baad022)
Supplement: baad022_Supp [file baad022_supp.zip › suppl_data/supplement material Fig S1-S5.docx]

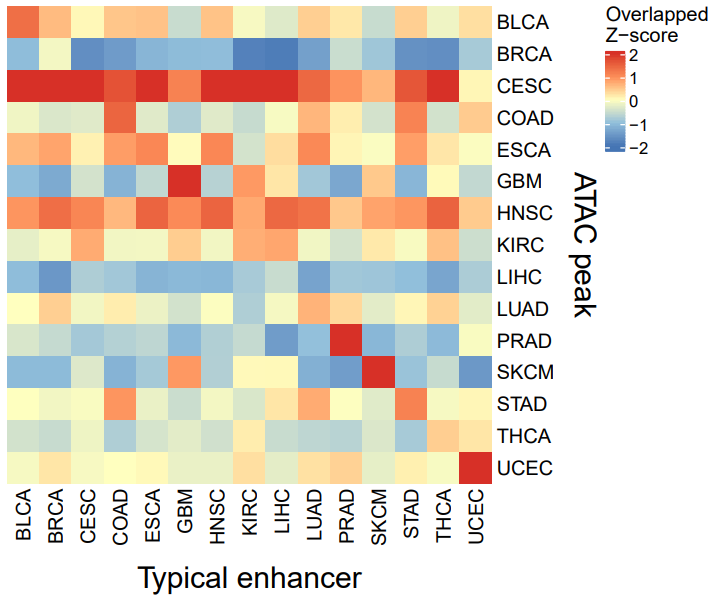


**Figure S1.** **Heatmap of Cell line typical enhancer overlap percentage with Primary tissue ATAC-Seq CARs.** The percentage is overlapped peaks dividing ATAC-Seq CARs, column was scaled for Z-score.


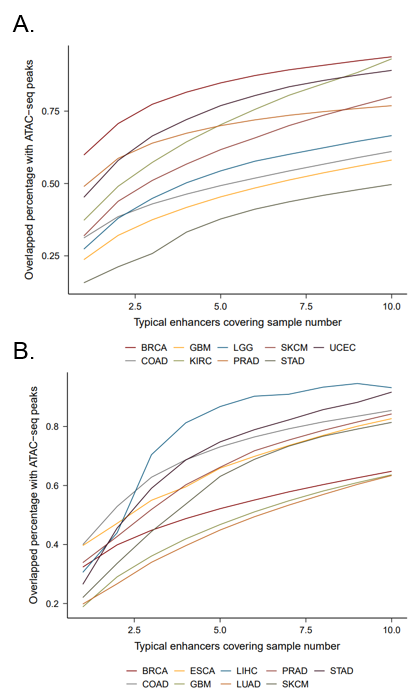


**Figure S2.** **Line chart of typical enhancer overlap percentage with Primary tissue ATAC-Seq CARs along the typical enhancer covered samples.** (A) typical enhancers from primary tissues overlapped with ATAC-Seq CARs. (A) typical enhancers from cell lines overlapped with ATAC-Seq CARs. Only cancer types with more than 10 samples are included.


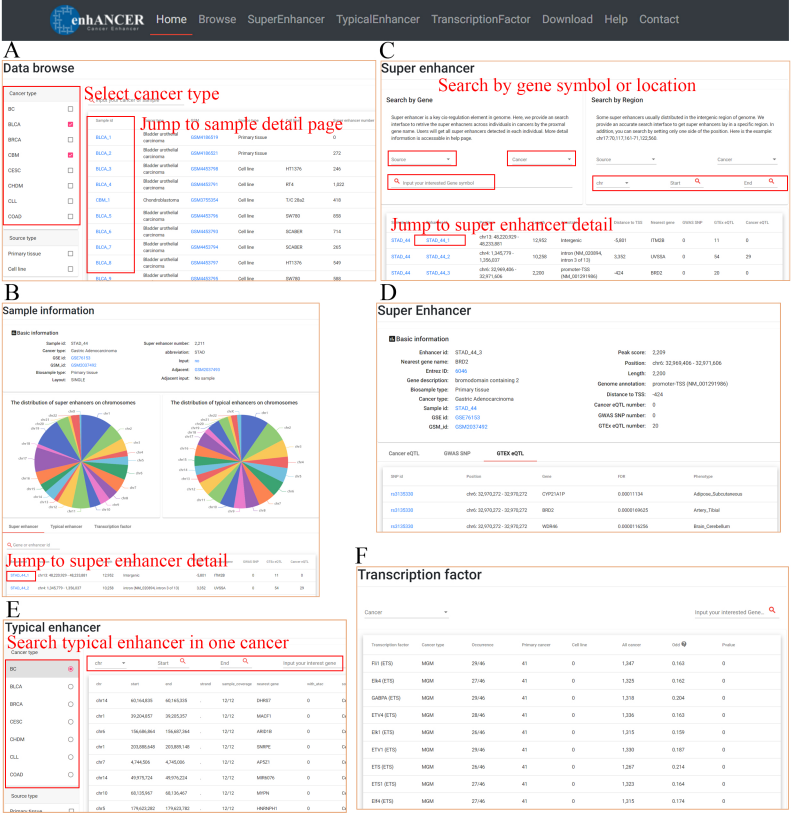


**Figure S3.** **The interface of CenhANCER.** (A) The data browser page. (B) The detail sample information page. (C) The super enhancer search page. (D) The detail super enhancer page. (E) The typical enhancer summary page. (F) The transcription factor summary page.


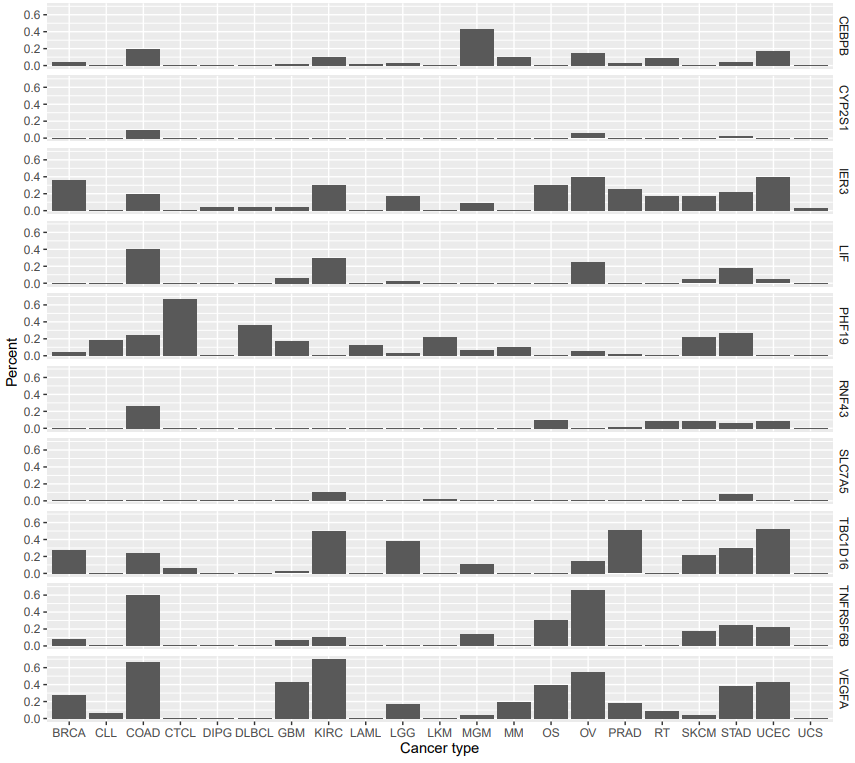


**Figure S4.** **The sample percentage of primary tissue.** The percentage are detected super enhancer samples in all samples for different cancer types in 10 genes. Only cancer types with more than 10 samples are included.


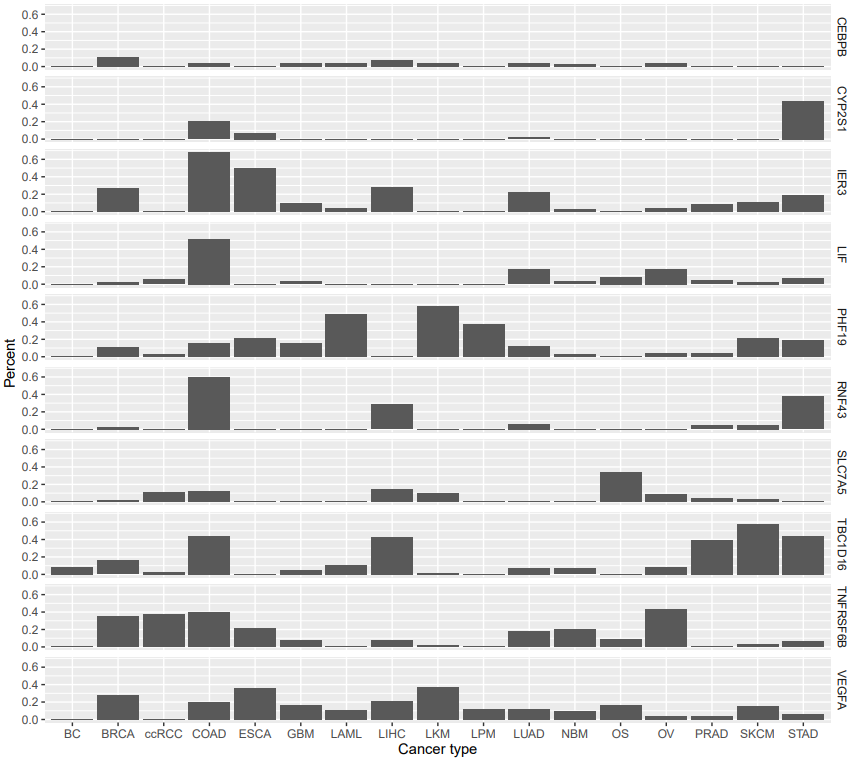


**Figure S5.** **The sample percentage of cell line.** The percentage are detected super enhancer samples in all samples for different cancer types in 10 genes. Only cancer types with more than 10 samples are included.
